# A Peer-Led Social Media Intervention to Improve Interest in Research Careers Among Urban Youth: Mixed Methods Study

**DOI:** 10.2196/16392

**Published:** 2020-05-14

**Authors:** Christianah Ogunleye, Jeanne M Farnan, Shannon K Martin, Audrey Tanksley, Samantha Ngooi, Laura Ruth Venable, Samantha Anderson, Jhonatan Marte, David O Meltzer, Vineet M Arora

**Affiliations:** 1 Pritzker School of Medicine University of Chicago Chicago, IL United States

**Keywords:** pipeline programs, minorities, underrepresented minorities, adolescents, teens, social media, peer led

## Abstract

**Background:**

Novel methods to boost interest in scientific research careers among minority youth are largely unexplored. Social media offers a unique avenue toward influencing teen behavior and attitudes, and can therefore be utilized to stimulate interest in clinical research.

**Objective:**

The aim of this study was to engage high-achieving minority youth enrolled in a science pipeline program to develop a targeted social media marketing campaign for boosting interest in clinical research careers among their peers.

**Methods:**

Students enrolled in the Training Early Achievers for Careers in Health program conducted focus groups in their communities to inform themes that best promote clinical research. They then scripted, storyboarded, and filmed a short video to share on social media with a campaign hashtag. Additionally, each student enrolled peers from their social circle to be subjects of the study. Subjects were sent a Career Orientation Survey at baseline to assess preliminary interest in clinical research careers and again after the campaign to assess how they saw the video, their perceptions of the video, and interest in clinical research careers after watching the video. Subjects who did not see the video through the online campaign were invited to watch the video via a link on the postsurvey. Interest change scores were calculated using differences in Likert-scale responses to the question “how interested are you in a career in clinical research?” An ordinal logistic regression model was used to test the association between watching a peer-shared video, perception of entertainment, and interest change score controlling for underrepresented minorities in medicine status (Black, American Indian/Alaska Native, Native Hawaiian, or Pacific Islander), gender, and baseline interest in medical or clinical research careers.

**Results:**

From 2014 to 2017, 325 subjects were enrolled as part of 4 distinct campaigns: #WhereScienceMeetsReality, #RedefiningResearch, #DoYourResearch, and #LifeWithoutResearch. Over half (n=180) of the subjects watched the video via the campaign, 227/295 (76.9%) found the video entertaining, and 92/325 (28.3%) demonstrated baseline interest in clinical research. The ordinal logistic regression model showed that subjects who viewed the video from a peer (odds ratio [OR] 1.56, 95% CI 1.00-2.44, *P*=.05) or found the video entertaining (OR 1.36, 95% CI 1.01-1.82, *P*=.04) had greater odds of increasing interest in a clinical research career. Subjects with a higher baseline interest in medicine (OR 1.55, 95% CI 1.28-1.87, *P*<.001) also had greater odds of increasing their interest in clinical research.

**Conclusions:**

The spread of authentic and relevant peer-created messages via social media can increase interest in clinical research careers among diverse teens. Peer-driven social media campaigns should be explored as a way to effectively recruit minority youth into scientific research careers.

## Introduction

Despite significant shifts in the demographic landscape of the United States over the past 50 years, minorities remain disproportionally underrepresented in medical practice and research [1]. As researchers often rely on personal experience and background to formulate questions, inadequate representation of underrepresented minority leaders in clinical research is an impediment to the adequate study of health conditions relevant to minority groups [2]. Attempts to alleviate this inequity typically focus on pipeline programs that directly target youth and adolescents. However, few studies have explored how to initially reach and recruit teens with low baseline interest or knowledge about science, technology, engineering, and mathematics (STEM) careers.

Enhanced interest in research careers has been previously described as a strong predictor of the “attitude” component of the theory of aligned ambition [3]. Conceptualized by Barbara Schneider, aligned ambition is a framework used to predict an adolescent’s future success in a desired career based upon their knowledge, behavior, and aforementioned attitude about said career path [4]. Because pipeline programs target recruitment at such an early stage, these programs influence all three aspects of aligned ambition, giving teens a more holistic picture of STEM fields. This type of pointed exposure aims to improve both the retention and persistence of minorities through scientific career paths [5].

In considering how to encourage interest in a scientific research career, the growing use of social media among adolescents offers new avenues toward influencing minority teens [6]. Both descriptive and experimental studies have supported targeted social marketing disseminated on the internet as an effective way to influence the health behaviors of teens. A 2014 systematic review found that 9 out of 10 online interventions reported significant improvements in some aspect of health behavior change [7]. From smoking cessation to health and sex education, peer-created social media campaigns are a powerful tool in engaging adolescents not only in the United States but also in international spheres [8-11]. However, no study has explored social media as a means to impact attitudes and behaviors regarding career interest.

Accordingly, the aim of this study was to engage high-achieving minority youth enrolled in a pipeline program with the Spreading Teen Research Inspired Videos to Engage Schoolmates (STRIVES) intervention. The purpose of STRIVES was to facilitate the creation of a video and peer-led social media campaign aimed at promoting interest in clinical research careers among their friends. We hypothesized that (1) a peer-shared video, compared to the same video shared by the investigative team, would be more effective in increasing interest in pursuing a career in research among peers of high-achieving minority youth enrolled in a pipeline program; and (2) videos that were perceived as entertaining would also be more effective in increasing interest in a clinical research career.

## Methods

### Study Design

#### Ethics

This study (IRB13-0848) was approved by the University of Chicago Biological Sciences Division Institutional Review Board (Chicago, IL, USA).

#### Setting

Training Early Achievers for Careers in Health (TEACH) is a pipeline initiative for rising high school juniors under the Collegiate Scholars Program, a partnership between the University of Chicago and Chicago Public Schools (CPS). The Collegiate Scholars Program is an intensive 3-year enrichment program designed to prepare talented high school students for academic success at the best colleges and universities. Starting in the summer after ninth grade, collegiate scholars select classes in literature, math, science, social sciences, and writing taught by University of Chicago faculty. Selection into this program is highly competitive, targeting students from ethnically and demographically underrepresented groups. Over 50% of collegiate scholars are underrepresented minorities (41% African American and 24% Latino/Hispanic), and 47.4% qualify to receive free or reduced lunch. Moreover, 41.56% of collegiate scholars will be first-generation college students [3].

Approximately 60 Collegiate Scholars Program students who expressed interest were selected as TEACH participants each year. These students were randomly assigned to one of two research groups: the clinical research group involving an immersive clinical research experience, or the field research group involving a more traditional basic science research program [3]. The 5-week clinical research summer program provided participants with several major experiences, including an introduction to the basics of clinical research through a classroom experience with faculty, an opportunity to work in a multitiered research team, and a hands-on research experience in a clinical environment performing patient interviews and physician surveys. The field research intervention consisted of science and nonscience lectures, a hands-on lab component led by PhD students, and field trips to local museums with high-quality science immersion.

#### Participant Recruitment

Each student enrolled in either the field or clinical research group recruited and provided contact information for approximately 10 “friends,” defined as peers from their general community. Peers were recruited though emails and postcards. The student that ultimately recruited the most peers was given 50 CPS learning hours and an Amazon gift card. We obtained written informed consent from each friend and a parent/guardian before dissemination of baseline assessments.

#### Spreading Teen Research Inspired Video to Engage Schoolmates

The STRIVES intervention within the clinical research program engaged students with the creation of a short video and the subsequent social media campaign aimed at promoting clinical research as a viable career among peers. To accomplish this, students had weekly STRIVES meetings with project managers, medical faculty and students, technology experts, and research assistants to guide them through the campaign process.

#### Focus Groups

We coached each cohort of students in the clinical research group to conduct one or two focus groups with 5-6 peers from the Collegiate Scholars Program to identify themes related to careers in clinical research as highlighted by the Appreciative Inquiry 4D Model. This model encompasses (1) Discovery, or identifying the best way to achieve the goal; (2) Dream, or imagining new means of achieving the goal; (3) Design, or how to operationalize a change to reach the goal; and (4) Destiny, or anticipating the best practice [12]. Focus groups were conducted at University of Chicago, which were moderated by the teens and transcribed by research assistants. Deductive analysis was utilized to identify themes as they related to the 4D model.

#### Video Creation

Using themes that emerged in focus groups about the perception of research careers and how to best engage peers, students discussed, storyboarded, and scripted preliminary video ideas for the STRIVES campaign. The investigative team reviewed the scripts for relevance and accuracy. Technology experts presented lectures to the students on video recording and editing tactics. Students recorded the videos on the University of Chicago Pritzker School of Medicine campus. Video editing, with assistance from project managers, was completed using Apple Final Cut Pro X (Cupertino, CA, USA).

#### Social Media Campaign

The investigative team trained the students in viral marketing techniques based on the usage of already established social networks to spread information. We taught the Activation Theory of Information Exposure [13], a social media marketing concept that highlights that the most effective messages are not only informative but also captivating [14]. Using the 5M Model [15] consisting of mission, market, money, message, and methodology, the students organized a strategy aimed at maximizing the influence and effectiveness of their social media campaign. We had the students complete a worksheet that related to the 5M model to craft a social media campaign. For example, each individual campaign had a message that correlated to the content of the video.

### Data Collection

Study subjects or “friends” completed an abbreviated online Career Orientation Survey following study enrollment to assess baseline knowledge, interest, and intent to pursue a variety of careers, including clinical research (see Multimedia Appendix 1). The Career Orientation Survey was originally developed and used in the Sloan Study of Youth and Social Development, a nationally representative longitudinal sample of American youth [16]. Adapted for the TEACH project, the screening instrument used in this study included standard and modified occupation survey items [3]. After the campaign, subjects once again completed the survey. Subjects who had not yet viewed the video through a peer-shared source by the time they received the postcampaign survey were given the opportunity to watch the video via the postsurvey. Of note, subjects who completed the postcampaign Career Orientation Survey in 2014 were not given the option to view the video via postsurvey; thus, all recorded responses were from subjects who viewed the video from a peer-disseminated source. We electronically sent friends who successfully submitted surveys an Amazon gift card and 5 CPS service learning hours. Study data were collected and managed using a Research Electronic Data Capture (REDCap) tool hosted at the University of Chicago [17].

The primary outcomes of interest were (1) perceptions of the video and (2) change in interest in pursuing a career in clinical research. Perceptions of the video were assessed by response on a 5-item Likert-type scale from “strongly disagree” to “strongly agree” in response to the statements “I found the video entertaining” and “I think this video is a good way to educate peers.” Career interest was assessed using the question “how interested are you in pursuing a career in clinical research?” with 5 Likert-type responses ranging from “definitely not interested” to “definitely interested.”

### Data Analysis

Descriptive and comparative statistics were used to analyze subjects’ demographic information and social media usage by year. An interest change score variable was calculated for each subject by taking the difference of Likert scores for researcher interest responses between the postcampaign and baseline Career Orientation Surveys.

An ordinal logistic regression was used to test the association between interest change score, a peer-shared vs survey-shared video, and perception of entertainment [18]. Model 1 controlled for (1) whether the subject was an underrepresented minority in medicine (ie, Black, Pacific Islander, Native American), (2) gender, (3) baseline interest in clinical research, (4) baseline interest in a medical career, and (5) whether the recruiting friend was in the clinical or field research group. Model 2 controlled for the same covariates listed above in addition to campaign year.

## Results

### Participant Characteristics

A total of 325 student-recruited subjects completed the pre and postcampaign Career Orientation Survey between 2014 and 2017. Of these subjects, 91.7% (n=298) watched the STRIVES video created by their respective year’s cohort from any source before completing the postcampaign Career Orientation Survey. Demographic characteristics (Table 1) varied by year. Among all subjects, the majority were female and 23.1% (75/325) were underrepresented minorities in the medical field (Black, American Indian/Alaska Native, Native Hawaiian or Pacific Islander).

### Perceptions About the Videos

**Figure 1:**
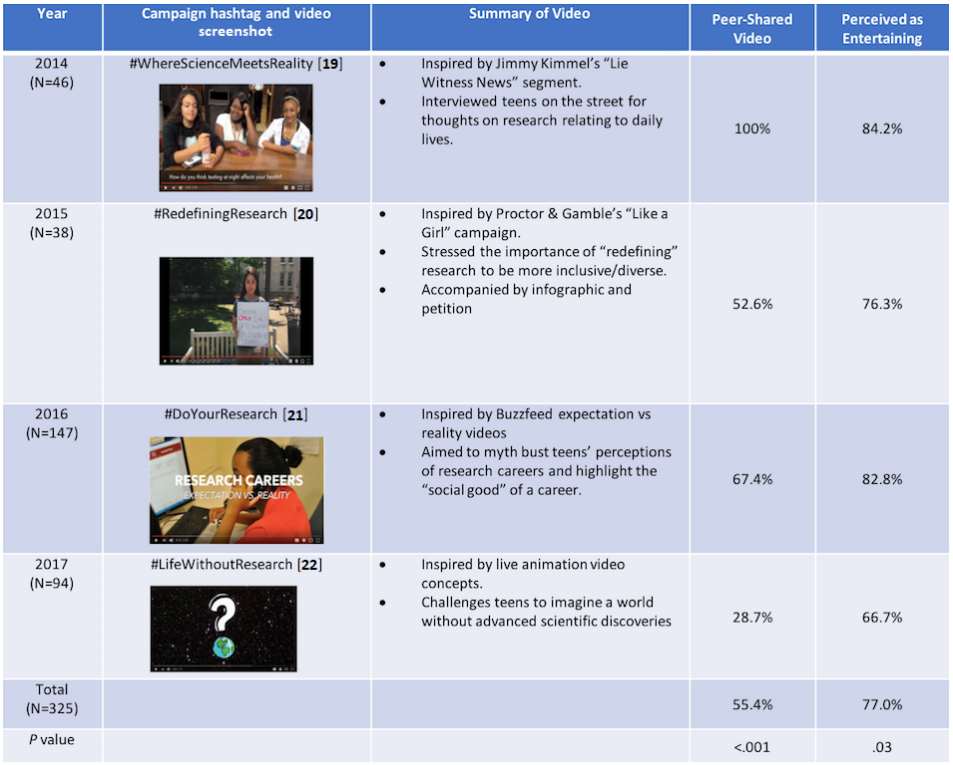
Spreading Teen Research Inspired Videos to Engage Schoolmates (STRIVES) video topics and summaries by year [19-22], including the percentage of subjects who watched the video through peer-disseminated sources during the social media campaign rather than through a link on the post-campaign Career Orientation Survey and the percentage of subjects who responded “agree” or “strongly agree” to the Likert-style question “I found the video entertaining” in the postcampaign Career Orientation Survey. *P* values were generated through Chi square analysis in STATA software.

A summary of STRIVES video topics by year, inspired by themes that arose in focus groups, are provided in Figure 1. Across all four cohorts, 55.4% (180/325) of the subjects watched a peer-shared video and 44.6% (145/325) watched a study-shared video. The majority of subjects from all cohorts had positive perceptions of their respective videos. For example, 76.9% (227/295) found the video entertaining and 81.1% (236/291) thought the video was a good way to educate peers. Among the subjects who watched a peer-shared video and completed the survey item, 80.9% (131/162) reported the video as entertaining, which was significantly higher (*P*=.04) than those that watched the video through the study (72.2%, 96/133). There was no difference in perception of whether videos were a good way to educate peers about careers based on the source of the video (study-shared 82% vs peer-shared 80%, *P*=.60).

**Table 1:** Demographic information of recruited friends by year.

Variable		Total (N=325)	2014 (N=46)	2015 (N=38)	2016 (N=147)	2017 (N=94)	*P* value^a^
**Gender, n (%)**							.03
	Male	100 (30.8)	12 (26.1)	10 (26.3)	52 (35.4)	26 (27.7)	
	Female	214 (65.8)	33 (71.7)	28 (73.7)	85 (57.8)	68 (72.3)	
	No Answer	11 (3.4)	1 (2.2)	0 (0)	10 (6.8)	0 (0)	
**Race, n (%)**							<.001
	American Indian/Alaska Native	3 (1.0)	0 (0)	0 (0)	3 (2.1)	0 (0)	
	Asian	78 (24.0)	15 (32.6)	10 (26.3)	24 (16.3)	29 (30.8)	
	Native Hawaiian or Other Pacific Islander	3 (1.0)	3 (6.5)	0 (0)	0 (0)	0 (0)	
	Black or African American	68 (20.9)	14 (30.4)	2 (5.3)	29 (19.7)	23 (24.5)	
	White	77 (23.6)	10 (21.8)	14 (36.8)	36 (24.5)	17 (18.1)	
	More than 1 race	29 (8.9)	0 (0)	2 (5.3)	16 (10.9)	11 (11.7)	
	Unknown/Prefer Not to Answer	67 (20.6)	4 (8.7)	10 (26.3)	39 (26.5)	14 (14.9)	
**Ethnicity, n (%)**							.03
	Hispanic	101 (31.1)	7 (15.2)	17 (44.7)	52 (35.4)	25 (26.6)	
	Non-Hispanic	219 (67.4)	39 (84.8)	21 (55.3)	91 (61.9)	68 (72.3)	
	No Answer	5 (1.5)	0 (0)	0 (0)	4 (2.7)	1 (1.1)	

^a^*P* values were generated through Chi square analysis in STATA (College Station, TX, USA) software.

### Interest in Clinical Research

**Figure 2:**
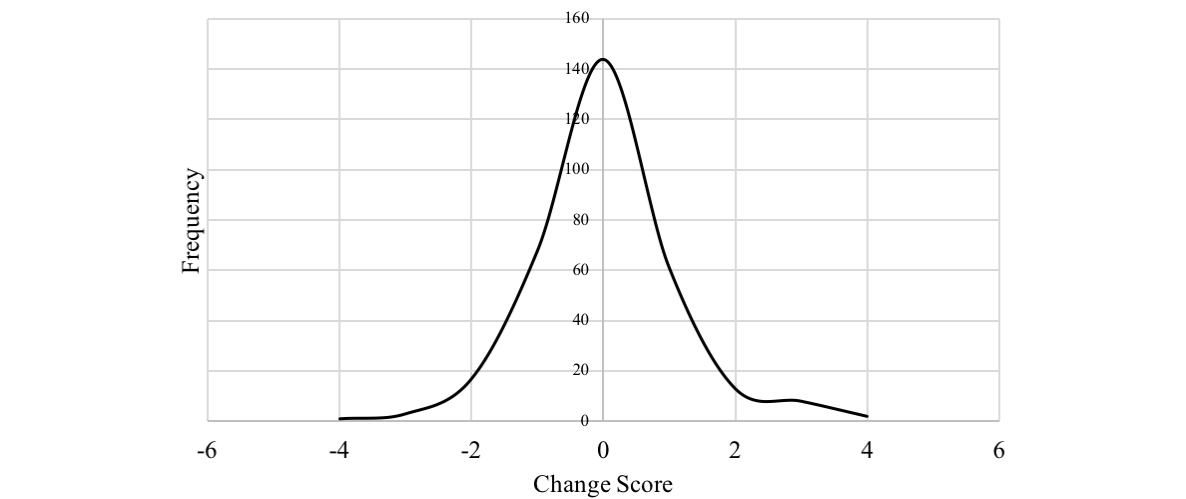
Change in career interest over time (2014-2017). Career interest was assessed using the Likert-type question, “how interested are you in pursuing a career in clinical research?” with responses ranging from “definitely not interested” to “definitely interested.” Positive interest was indicated by a response of “definitely interested” or “very interested.” The change score variable was calculated by taking the difference of Likert scores for researcher interest responses between the postcampaign and baseline Career Orientation Surveys.

Before being exposed to the STRIVES campaign, 28.3% (92/325) of the subjects showed interest in a clinical research career compared to 25.8% (84/325) after the STRIVES campaign. Change scores were approximately normally distributed (Figure 2). Of the study subjects, 45.4% (144/317) did not change their interest in a clinical research career after watching the STRIVES video, 28.1% (89/317) expressed less interest, and 26.5% (84/317) showed greater interest. A significantly higher percentage of subjects who watched a peer-shared video showed postcampaign interest than those who watched a study-shared video (58/180, 32.2% and 30/145, 20.7%, respectively; *P*=.04).

In the multivariate ordinal logistic regression (Table 2), watching a peer-shared video vs a study-shared video was significantly associated with increased odds of higher interest in a clinical research career. Those who found the video to be entertaining also had greater odds of increased interest in clinical research. In addition, subjects who had higher baseline interest in medicine were associated with greater odds of increased interest in clinical research, whereas higher baseline interest in clinical research was associated with lower odds of showing increased interest. Gender, underrepresented minority in medicine status, and whether the recruiting friend was in the clinical research program were not significantly associated with an increasing interest in a clinical research career.

In model 2 of the multivariate ordinal logistic regression, controlling for campaign year, those who found the video to be entertaining had greater odds of increased interest in clinical research, whereas there was no association based on whether the video was peer-shared or study-shared. Similar to model 1, subjects who had higher baseline interest in medicine were associated with greater odds of increased interest in clinical research, whereas a higher baseline interest in clinical research was associated with lower odds of showing increased interest after watching the video. Watching a peer-shared video, gender, campaign year, underrepresented minority in medicine status, and whether the recruiting friend was in the clinical research program were not significantly associated with a change in clinical research interest.

**Table 2:** Ordinal logistic regression testing the association between predictors and change in clinical research interest.

Variable (N=287)	Model 1			Model 2^a^			
	Odds ratio (SE)	95% CI	*P* value		Odds ratio (SE)	95% CI	*P* value
Peer-shared video^b^	1.56 (0.36)	1.00-2.44	.05		1.50 (0.36)	0.94-2.41	.09
Perceived video as entertaining	1.36 (0.20)	1.01-1.82	.04		1.35 (0.20)	1.00-1.82	.04
Baseline interest in clinical research	0.32 (0.04)	0.25-0.41	<.001		0.32 (0.04)	0.25-0.41	<.001
Baseline interest in medicine	1.55 (0.15)	1.28-1.87	<.001		1.55 (0.15)	1.29-1.87	<.001
Recruited by clinical research student^c^	1.08 (0.25)	0.69-1.69	.73		1.09 (0.25)	0.70-1.71	.70
Underrepresented minority in medicine^d^	0.88 (0.25)	0.50-1.53	.65		0.89 (0.25)	0.51-1.55	.67
Male gender	0.95 (0.23)	0.59-1.52	.82		0.95 (0.23)	0.59-1.52	.82

^a^Model 2 controlled for campaign year in addition the listed variables.

^b^Subjects watched the video from a peer-shared source vs a survey-shared source (ie, through a link provided on the postcampaign Career Orientation Survey).

^c^Subjects were recruited by friends participating in the clinical research program vs friends participating in the field research program.

^d^Underrepresented minority in medicine characterized as Hispanic, Black, Pacific Islander, or Native American.

## Discussion

The results of this study suggest that peer-led social media campaigns paired with short, entertaining videos are an effective way to increase interest in clinical research careers among peers. Specifically, we found that videos shared via peers on social media were more effective in improving interest in clinical research compared to the same video watched through a link provided by the investigative team. Videos that subjects perceived as entertaining were also more likely to be associated with an increase in interest in clinical research careers among subjects.

Approximately half (55.4%, 180/325) of our study subjects viewed a STRIVES video through an online social media source as shared by a peer in the TEACH program. It is likely that these subjects who came across the videos on social media were already well-embedded within the social network of the students who not only created the videos but played an acting role within them. This results in a sense of perceived familiarity with the content, which in turn allowed the subjects to be more positively influenced by the videos [6]. These findings are consistent with other works showing how peer-to-peer interactions among adolescents are key to influencing teen cognition and behavior [23]. In fact, this strategy is often evoked by corporate marketing campaigns in the age of digital media, particularly when utilizing celebrity sponsorships or social media influencers with whom the targeted audiences feel more connected to [24]. Information received from peers is deemed to be not only credible but also relatable, particularly when paired with the interactivity of social media [25]. This influential effect of peer communication on adolescents has been shown to be even stronger among both female and underrepresented minority teens, thereby strengthening the efficacy of social media campaigns in engaging minority teens [26].

Subjects who watched a peer-shared video were significantly more likely to find the video entertaining and were also more likely to report increased interest in clinical research. This correlation can be explained within the context of entertainment value being crucial to how messages are perceived and processed. Content that is deemed to be amusing becomes more compelling to the viewer, resulting in increased internalization of the message and more engagement with the post [27]. Social media algorithms function in such a way that posts with the most interactions become more visible to those outside of the intended network [28]. This increase in visibility then gives way to a larger spread of the post and eventually its influence. In essence, entertaining videos are more likely to be shared, subsequently increasing the visibility and engagement, leading to a larger effect size.

Future considerations for interventions such as STRIVES require exploration of how these efforts translate into pipeline program enrollment. Campaigns and other social marketing endeavors act to change attitudes (ie, interest in scientific research careers), whereas more immersive counterparts such as pipeline programs are needed to influence the action of pursuing clinical research as a career. Essentially, social media campaigns are an important step toward “priming the pump.” Forthcoming efforts to recruit teens to clinical research pipeline programs should leverage these findings in an attempt to bolster the number of interested applicants. Similar efforts are worth testing among college students and medical students given concerns of the physician-scientist pipeline.

The primary limitation of this study was the inability to randomize subjects into peer-shared vs study-shared video groups. This led to significant variability in the number of subjects exposed to peer-shared videos across the campaign years. For example, in 2014, 100% of subjects who completed the postsurvey viewed the STRIVES campaign from a peer-shared source. This inherently conflated associations between the two groups as there was no comparison available for peer-shared viewers that year. This likely attributed to the video source no longer being a significant predictor of changing interest in research when controlling for campaign year as seen in model 2 of the ordinal logistic regression.

Overall, this study demonstrated that peer-shared videos that are perceived as entertaining are significantly associated with increasing interest in a clinical research career among peers of high-achieving minority youth in a scientific research pipeline program. Authentic and relevant peer-driven messages have the potential to engage and activate minority youth, thus “priming the pump” into clinical research pipeline programs. The ultimate hope is that early exposure will translate into fruitful careers that will help diversify the scientific research workforce.

## Appendix

Multimedia Appendix 1 The Career Orientation Survey (COS) was originally developed and used in the Sloan Study of Youth and Social Development, a nationally representative longitudinal sample of American youth. This survey was sent to study subjects at baseline and again after the social media campaign to gain insight on social media usage and career alignment.

## Abbreviations

CPS: Chicago Public Schools
REDCap: Research Electronic Data Capture
STEM: science, technology, engineering, and mathematics
STRIVES: Spreading Teen Research Inspired Videos to Engage Schoolmates
TEACH: Training Early Achievers for Careers in Health
