# Supplementary material for: A Peer-Led Social Media Intervention to Improve Interest in Research Careers Among Urban Youth: Mixed Methods Study
Source: JMIR Med Educ. 2020 May 14;6(1):e16392. doi: 10.2196/16392 (PMC7256755; doi:10.2196/16392)
Supplement: Multimedia Appendix 1 [file mededu_v6i1e16392_app1.pdf]

Strives Post Survey Friends

Please complete the survey below. Once we have received confirmation that you have completed the survey you will receive an email containing your $5 gift card and Service Learning Hour letter.

Thank you!

Did you see a video from one of your friends who was in the video?
- ◯ Yes
- ◯ No

If you have not seen the video, please watch it here in a new window before proceeding:

Did you watch the video above?
- ◯ Yes
- ◯ No

How much do you agree with the following statements?

	Strongly Disagree	Disagree	Neutral	Agree	Strongly Agree
I found the video entertaining	◯	◯	◯	◯	◯
The video has changed my interest in a clinical research career in a positive way	◯	◯	◯	◯	◯
The video has changed my interest in a clinical research career in a negative way	◯	◯	◯	◯	◯
I think videos are a good way to reach my peers about career options	◯	◯	◯	◯	◯

Where did you watch the video?
- ◯ Facebook
- ◯ Twitter
- ◯ Email
- ◯ YouTube
- ◯ ScienceLife blog
- ◯ CHeSS website
- ◯ This survey
- ◯ Other

Other: ______________________________

How often do you use social media (or online communities to electronically share information, idea, personal messages, and other content (such as Facebook, Twitter, You Tube, etc)
- ◯ Almost Everyday
- ◯ 3 - 4 times a week
- ◯ 1 - 2 times a week
- ◯ 1 - 2 times a month
- ◯ Never / rarely

Please rank the social media you use most often (with 1 being least used and 6 being most used)

	1	2	3	4	5
Twitter	○	○	○	○	○
Facebook	○	○	○	○	○
Instagram	○	○	○	○	○
Tumblr	○	○	○	○	○
Snapchat	○	○	○	○	○

Other (please specify) __________________________________

What devices do you use to view social media? (check all that apply)
- ☐ Smartphone
- ☐ Tablet/iPad
- ☐ Computer

List a job that you WOULD LIKE TO DO when you are older __________________________________

How sure you are that you will have the job of [like1_post] when you are older?
- ○ Unsure
- ○ Pretty Sure
- ○ Very Sure

How much education do you think is necessary for the job of [like1_post]
- ○ Less than a high school diploma
- ○ High school diploma only
- ○ 2-year college / vocational certificate
- ○ College degree
- ○ Master's degree
- ○ PhD., M.D. or other equivalent professional degree
- ○ Special training beyond PhD or M.D.

Enter the yearly income you think you would earn after working as a [like1_post] for 5 years __________________________________

On a scale of 1-100, how much do you think people would look up to you if you had the job of [like1_post]? 100 means people would look up to you the most. 1 means people wouldn't look up to you at all. __________________________________

List another job that you WOULD LIKE TO DO when you are older __________________________________

How sure are you that you will have the job of [like2_post] when you are older?
- ○ Unsure
- ○ Pretty Sure
- ○ Very Sure

How much education do you think is necessary for the job of [like2_post]?
- ○ Less than a high school diploma
- ○ High school diploma only
- ○ 2-year college / vocational certificate
- ○ College degree
- ○ Master's degree
- ○ PhD., M.D. or other equivalent professional degree
- ○ Special training beyond PhD or M.D.

Enter the yearly income you think you would earn after working as a [like2_post] for 5 years __________________________________

On a scale of 1-100, how much do you think people would look up to you if you had the job of [like2_post]? 100 means people would look up to you the most. 1 means people wouldn't look up to you at all. __________________________________

The following questions will ask you about jobs you THINK YOU WILL DO. If the jobs you think you will do are the same as the jobs you would like to do (from the previous set of questions) be sure to list the jobs in both places.

List a job that you THINK YOU WILL DO when you are older __________________________________

How much education do you think is necessary for the job of [willdo1_post]?

- ◯ Less than a high school diploma
- ◯ High school diploma only
- ◯ 2-year college / vocational certificate
- ◯ College degree
- ◯ Master's degree
- ◯ PhD., M.D. or other equivalent professional degree
- ◯ Special training beyond PhD or M.D.

Enter the yearly income you think you would earn after working as a [willdo1_post] for 5 years __________________________________

On a scale of 1-100, how much do you think people would look up to you if you had the job of [willdo1_post]? 100 means people would look up to you the most. 1 means people wouldn't look up to you at all. __________________________________

List another job that you THINK YOU WILL DO when you are older __________________________________

How much education do you think is necessary for the job of [willdo2_post]?

- ◯ Less than a high school diploma
- ◯ High school diploma only
- ◯ 2-year college / vocational certificate
- ◯ College degree
- ◯ Master's degree
- ◯ PhD., M.D. or other equivalent professional degree
- ◯ Special training beyond PhD or M.D.

Enter the yearly income you think you would earn after working as a [willdo2_post] for 5 years __________________________________

On a scale of 1-100, how much do you think people would look up to you if you had the job of [willdo2_post]? 100 means people would look up to you the most. 1 means people wouldn't look up to you at all. __________________________________

How much information have the following sources given you about the FIRST job you THINK you will do ([willdo1])?

	1 None	2	3 Some	4	5 A Lot
Television	◯	◯	◯	◯	◯
School Classes	◯	◯	◯	◯	◯
A teacher	◯	◯	◯	◯	◯
A school counselor	◯	◯	◯	◯	◯
Newspaper/magazine/books	◯	◯	◯	◯	◯
Talking with friends	◯	◯	◯	◯	◯
Your family	◯	◯	◯	◯	◯
Your job(s)	◯	◯	◯	◯	◯
Internet	◯	◯	◯	◯	◯
Other	◯	◯	◯	◯	◯

Let's look at the jobs you THINK YOU WILL DO (the ones you just answered questions about). Are you doing anything now that is related to those jobs? For example, if you wanted to be an insurance agent, you might be helping your father at his insurance office.

Do you do anything now that relates to the job of [willdo1_post]?
◯ Yes
◯ No

Please describe what you do.
____

Do you do anything now that relates to the job of [willdo2_post]?
◯ Yes
◯ No

Please describe what you do.
____

How interested are you in pursuing a career as a business person?
◯ Definitely not interested
◯ Not Interested
◯ Somewhat Interested
◯ Very interested
◯ Definitely interested
◯ Don't Know

How interested are you in pursuing a career as a lawyer?
◯ Definitely not interested
◯ Not Interested
◯ Somewhat Interested
◯ Very interested
◯ Definitely interested
◯ Don't Know

How interested are you in pursuing a career as a medical doctor?
◯ Definitely not interested
◯ Not Interested
◯ Somewhat Interested
◯ Very interested
◯ Definitely interested
◯ Don't Know

How interested are you in pursuing a career in clinical research?

◯ Definitely not interested
◯ Not Interested
◯ Somewhat Interested
◯ Very interested
◯ Definitely interested
◯ Don't Know
